# Early kidney injury predicts disease progression in patients with COVID-19: a cohort study

**DOI:** 10.1186/s12879-021-06576-9

**Published:** 2021-09-27

**Authors:** Tingting Xia, Wenjing Zhang, Yu Xu, Bin Wang, Zhiquan Yuan, Na Wu, Ying Xiang, Chengying Li, Yifan Shan, Weijia Xie, Youhao Wang, Yao Zhang, Li Bai, Yafei Li

**Affiliations:** 1 Department of Epidemiology, College of Preventive Medicine, Army Medical University (Third Military Medical University), NO.30 Gaotanyan Street, Chongqing, 400038 People’s Republic of China; 2 Department of Respiratory and Critical Care Medicine, the Second Affiliated Hospital of Army Medical University, Chongqing, 400037 People’s Republic of China

**Keywords:** Coronavirus disease 2019, Severe respiratory syndrome coronavirus 2, Kidney injury, Disease progression

## Abstract

**Background:**

The receptor of severe respiratory syndrome coronavirus 2 (SARS-CoV-2), angiotensin-converting enzyme 2, is more abundant in kidney than in lung tissue, suggesting that kidney might be another important target organ for SARS-CoV-2. However, our understanding of kidney injury caused by Coronavirus Disease 2019 (COVID-19) is limited. This study aimed to explore the association between kidney injury and disease progression in patients with COVID-19.

**Methods:**

A retrospective cohort study was designed by including 2630 patients with confirmed COVID-19 from Huoshenshan Hospital (Wuhan, China) from 1 February to 13 April 2020. Kidney function indexes and other clinical information were extracted from the electronic medical record system. Associations between kidney function indexes and disease progression were analyzed using Cox proportional-hazards regression and generalized linear mixed model.

**Results:**

We found that estimated glomerular filtration rate (eGFR) and creatinine clearance (Ccr) decreased in 22.0% and 24.0% of patients with COVID-19, respectively. Proteinuria was detected in 15.0% patients and hematuria was detected in 8.1% of patients. Hematuria (HR 2.38, 95% CI 1.50–3.78), proteinuria (HR 2.16, 95% CI 1.33–3.51), elevated baseline serum creatinine (HR 2.84, 95% CI 1.92–4.21) and blood urea nitrogen (HR 3.54, 95% CI 2.36–5.31), and decrease baseline eGFR (HR 1.58, 95% CI 1.07–2.34) were found to be independent risk factors for disease progression after adjusted confounders. Generalized linear mixed model analysis showed that the dynamic trajectories of uric acid was significantly related to disease progression.

**Conclusion:**

There was a high proportion of early kidney function injury in COVID-19 patients on admission. Early kidney injury could help clinicians to identify patients with poor prognosis at an early stage.

**Graphic abstract:**

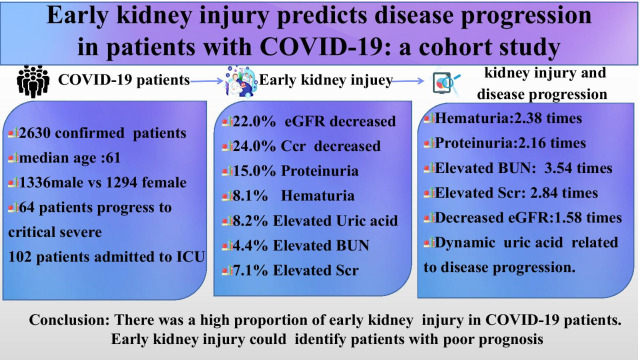

**Supplementary Information:**

The online version contains supplementary material available at 10.1186/s12879-021-06576-9.

## Background

Since December 2019, Coronavirus Disease 2019 (COVID-19) outbreak, caused by severe respiratory syndrome coronavirus 2 (SARS-CoV-2), has become a worldwide pandemic and public health emergency [1]. Respiratory system injury is the principal clinical manifestation of COVID-19, and acute respiratory distress syndrome is one of life-threatening complications [2]. Most studies have focused on the clinical characteristics of pneumonia caused by COVID-19. However, a significant fraction of patients with COVID-19 display abnormalities in kidney function [3].

Existing studies have shown that SARS-CoV-2 virus may specifically attack the kidney, causing acute kidney injury (AKI) [4]. However, the evidence of kidney injury in COVID-19 patients remained controversial. Studies reported that acute kidney injury was a frequent condition in critically ill patients that has been associated with substantial morbidity and mortality [2, 5]. However, a study with an analysis of 116 hospitalized patients with COVID-19 in a single hospital showed that SARS-CoV-2 infection does not significantly cause AKI [6]. Indeed, the significance of AKI in patients with COVID-19 remained uncertain due to a lack of clear and operable diagnostic criteria for AKI in most medical reports. Previous studies mainly focused on COVID-19 patients superimposed acute kidney injury. However, the early decline of renal function, usually evaluated by abnormal estimated glomerular filtration rate (eGFR) and endogenous creatinine clearance rate (Ccr), could occur prior to acute kidney injury [7]. At present, few studies have explored the relationship between early kidney function indexes and disease progression of COVID-19, especially in the aspects of the dynamic changes of the early kidney function indexes. Thus, we conducted this large hospital-based cohort study to address the association of early kidney injury and COVID-19 disease progression.

## Methods

### Study design and subjects

**Fig. 1:**
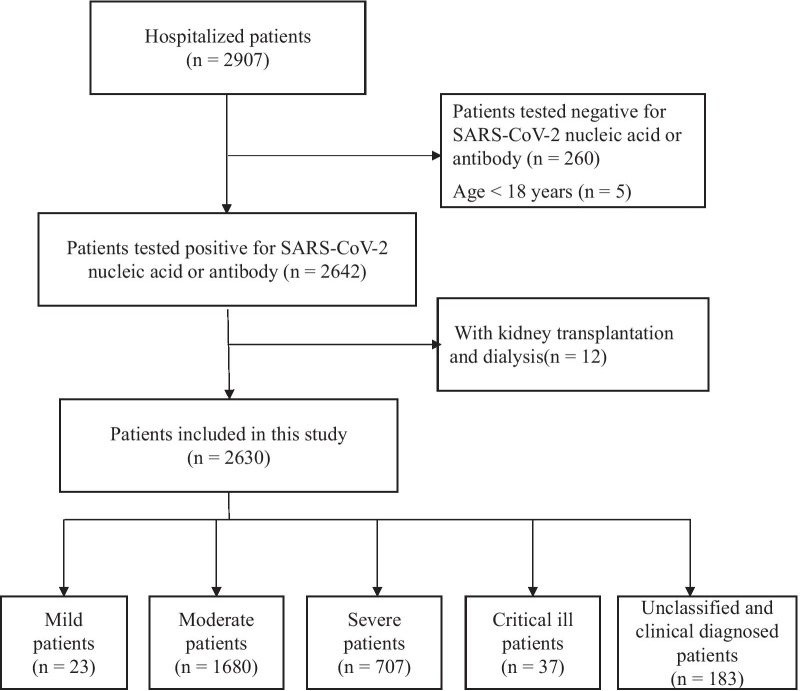
Flow chat of patient inclusion

This was a retrospective hospital-based cohort study. COVID-19 patients were enrolled from Huoshenshan Hospital, which was one of the largest special hospital of COVID-19, in Wuhan, China. Patients were enrolled from February to April 2020 with the inclusion criteria: at least 18 years old and confirmed with SARS-CoV-2 infection based on positive nucleic acid or antibody detection. Patients with a history of kidney diseases, dialysis or kidney transplantation were excluded from this study (Fig. 1). This study was approved by the Ethics Committee of Huoshenshan Hospital.

According to “Diagnosis and Treatment Protocol for Novel Coronavirus Infection-Induced Pneumonia (Version 7)” published by the National Health Commission of China [8], patients were categorized into four clinical types based on the severity assessment of COVID-19: mild, moderate, severe and critical cases. The discharge criteria were defined as the following conditions: (1) body temperature returned to normal for at least three days; (2) respiratory symptoms improved significantly; (3) pulmonary imaging showed significant absorption of inflammation; (4) nucleic acid test was negative for two consecutive times on respiratory tract samples, with sampling interval of at least 24 h.

### Data collection

A standardized uniform form was used to collect data. Demographic information (age and sex), clinical symptoms (from the onset to admission), laboratory measurements, medical treatment (antibiotic, antiviral, traditional Chinese medicine, oxygen therapy, glucocorticoid therapy, respiratory support) and outcomes were extracted through the electronic medical record system. Dynamic kidney laboratory parameters included Scr (Serum creatinine), BUN (blood urea nitrogen), uric acid, eGFR (estimated glomerular filtration rate), and Ccr (creatinine clearance rate) on admission, mid-term hospitalization and the final test before discharge were collected at the same time. Other laboratory measurements included: complete blood count, blood chemical analysis, coagulation testing, hypersensitive C-reactive protein (CRP), brain natriuretic peptide (BNP), procalcitonin, myocardial infarction kinase, routine urine and stool check results. Neutrophil-to-lymphocyte ratio (NLR, neutrophil count divide by lymphocyte count) was used to represent inflammatory markers on admission. The inflammatory markers cut-off point was determined using the X-tile software (version 3.50). All data were independently processed and reviewed by two researchers (Yu Xu and Bin Wang).

### Kidney function indexes

Serum creatinine (Scr), blood urea nitrogen (BUN), and uric acid (UA) were measured. Additional file 1: Table S1 showed the upper limit of normal values of these indexes. Proteinuria and hematuria were defined as positive in urine with semiquantitative urinalysis.

eGFR was calculated based on the Chronic Kidney Disease Epidemiology Collaboration (CKD-EPI serum creatinine) formula, for it was considered more precise and less biased than MDRD equation recommended by National Institute for Health and Care Excellence Clinical Practice Guideline [9].

Male ≤ 80 years old : eGFR=141×[Scr (umol/L) /80]^¯0.411^×0.993age (year-old) Male > 80 years old : eGFR=141×[Scr (umol/L) /80]^¯1.328^×0.993age (year-old) Female ≤ 62 years old : eGFR=144×[Scr (umol/L) /62]^¯0.329^×0.993age (year-old)  Female > 62 years old: eGFR=144×[Scr (umol/L) /62]^¯1.209^×0.993age (year-old) Ccr was calculated according to Cockcroft's formula as follow. Ccr = [140 − Age (year-old)] × weight (kg) ÷ [0.818 × Scr (umol/L)] (female × 0.85).

In this study, early kidney injury was defined as eGFR < 90 min*1.73 m^2^ or Ccr < 80 min*1.73 m^2^. Acute kidney injury was determined according to Kidney Disease: Improving Global Outcomes (KDIGO) [10].

### Outcomes

The primary outcome was disease progression, which was defined by the following criteria: (1) Severity of COVID-19 changed from mild/moderate into severe/critical during hospital stay. (2) Death. (3) Transfer to ICU after initial hospital admission (i.e. more than 24 h of admission).

### Statistical analyses

Categorical variables were described as frequency and percentage. Continuous variables were described as median and interquartile range (IQR) values. A Cox proportional-hazards regression analysis was used to assess the associations between early kidney function indexes and outcomes. Three methods (entering, forward and backward for likelihood ratio test) in multivariable Cox regression analysis were used to screen potential confounders including age at diagnosis, gender, smoking, clinical types, comorbidity, laboratory tests and treatment. The significant variables were retained as covariates for adjustment in subsequent multivariable Cox regression analysis by entering method. Variance inflation factor (VIF) and the condition index (CI) were used to identify collinearity among the covariates. VIF > 5 and condition index > 10 were considered as collinearity and can not entered the multivariate Cox regression analysis. Rude and adjusted hazard ratios (HRs) and 95% confidence intervals (CIs) were calculated. A generalized linear mixed model analysis was performed to assess the association between dynamic variation of kidney function indexes and outcomes. Statistical analyses were performed using SPSS version 25.0. A two-tailed P-value < 0.05 was considered statistically significant.

## Results

### Demographical and clinical information of patients

A total of 2630 hospitalized patients with confirmed COVID-19 were finally enrolled in this cohort study. The age ranged from 18 to 100 years (median 61; IQR 50–68). The median days from the illness onset to admission were 4 days (IQR 0–8). There were 1053 (40.0%) patients who reported more than three symptoms on admission. According to the severity of disease on admission, patients were classified into four clinical types: mild (23, 0.9%), moderate (1680, 63.9%), severe (707, 26.9%), and critically ill (37, 1.4%); 7% (183) patients were unclassified. Sixty-four patients progressed to critical or severe. 102 patients admitted to ICU during hospitalization. By the end of April 13th, 2539 (96.5%) patients were discharged, 62 (2.4%) patients died, and 29 (1.1%) patients were lost to follow-up (Table 1).

**Table 1:** Demographic and clinical characteristics of COVID-19 patients

Characteristics	All patients (n = 2630)	Mild patients (n = 23)	Moderate patients (n = 1680)	Severe patients (n = 707)	Critically ill patients (n = 37)	Unclassified and clinically diagnosed patients (n = 183)
Age, median (IQR), y						
	61 (50–68)	49 (29–63)	58 (48–67)	64 (56–72)	74 (56.5–81)	61 (51–68)
Gender						
Male	1336 (50.8%)	9 (39.1%)	836 (49.8%)	371 (52.5%)	25 (67.6%)	95 (51.9%)
Female	1294 (49.2%)	14 (60.9%)	844 (50.2%)	336 (47.5%)	12 (32.4%)	88 (48.1%)
Time from illness onset to hospital admission, days						
	4 (0–8)	0 (0–4.5)	4 (1–9)	3 (0–7)	4 (0–8.5)	4 (0–8)
Smoking history						
Yes	186 (7.1%)	3 (13.0%)	116 (6.9%)	51 (7.2%)	2 (5.4%)	14 (7.7%)
Exposure history						
Yes	438 (16.7%)	8 (34.8%)	294 (17.5%)	95 (13.4%)	3 (8.1%)	38 (20.8%)
Symptoms						
Fever	1925 (73.2%)	8 (34.8%)	1219 (72.6%)	532 (74.7%)	25 (67.6%)	141 (77.0%)
Cough	1860 (70.7%)	8 (34.8%)	1181 (70.3%)	517 (72.6%)	28 (75.7%)	126 (68.9%)
Fatigue	1424 (54.1%)	9 (39.1%)	897 (53.4%)	413 (58.0%)	20 (54.1%)	85 (46.4%)
Asthma	1173 (44.6%)	4 (17.4%)	699 (41.6%)	378 (53.1%)	20 (54.1%)	72 (39.3%)
Myalgia	788 (30.0%)	3 (13.0%)	498 (29.7%)	214 (30.1%)	12 (32.4%)	61 (33.3%)
Other symptoms	712 (27.1%)	5 (21.7%)	452 (26.9%)	202 (28.4%)	7 (18.9%)	46 (25.1%)
Symptoms counts (> 3)	1053 (40.0%)	4 (17.4%)	645 (38.4%)	315 (44.2%)	19 (51.4%)	70 (38.3%)
Comorbidities						
Hypertension	848 (32.3%)	6 (26.1%)	465 (27.7%)	307 (43.1%)	17 (45.9%)	53 (29.0%)
CAD	184 (7.0%)	1 (4.3%)	92 (5.5%)	73 (10.3%)	4 (10.8%)	14 (13.1%)
Heart failure	11 (0.4%)	0 (0%)	4 (0.2%)	4 (0.6%)	2 (5.4%)	1 (0.5%)
Other cardio disease	87 (3.3%)	0 (0%)	35 (2.1%)	33 (4.6%)	8 (21.6%)	11 (6.0%)
Respiratory distress	10 (0.5%)	0 (0%)	0 (0%)	2 (0.3%)	7 (18.9%)	1 (0.5%)
COPD	24 (0.9%)	0 (0%)	11 (0.7%)	8 (1.1%)	3 (8.1%)	2 (1.1%)
Respiratory failure	28 (1.1%)	0 (0%)	0 (0%)	11 (1.5%)	14 (37.8%)	3 (1.6%)
Chronic kidney disease	31 (1.2%)	0 (0%)	17 (1.0%)	6 (0.8%)	3 (8.1%)	5 (2.7%)
Tumor history	27 (1.0%)	0 (0%)	14 (0.8%)	7 (1.0%)	3 (8.1%)	3 (1.6%)
Diabetes	385 (14.6%)	0 (0%)	214 (12.7%)	139 (19.5%)	8 (21.6%)	24 (13.4%)
Respiratory comorbidity	153 (5.8%)	0 (0%)	66 (3.9%)	47 (6.6%)	24 (64.9%)	16 (8.7%)
Other comorbidities	626 (23.8%)	6 (26.1%)	369 (23.0%)	200 (30.2%)	14 (42.4%)	37 (20.2%)
Comorbidity counts (> 3)	96 (3.7%)	0 (0%)	42 (2.5%)	40 (5.6%)	8 (21.6%)	6 (3.3%)
Maximum temperature, ℃ (IQR)	37.1 (36.9–37.4)	37.0 (36.8–37.8)	37.0 (36.9–37.3)	37.2 (36.9–37.8)	37.5 (37.1–38.7)	37.1 (36.9–37.5)
Respiratory rate, breath per min	20 (19–21)	20 (19–22)	20 (19–21)	20 (19–22)	23 (19–34)	20 (20–21)
Systolic pressure, mmHg	130 (120–140)	123 (116–132)	129 (120–139)	130 (120–143)	130 (117–143)	128 (117–138)
Diastolic pressure, mmHg	80 (74–88)	77 (70–86)	80 (74–88)	80 (74–88)	81 (70–89)	80 (72–86)
Pluses, times/min	85 (78–96)	84 (74–96)	84 (78–95)	86 (78–98)	98 (89–107)	84 (77–90)
Therapy						
Antibiotics	840 (31.9%)	3 (13.0%)	441 (26.3%)	294 (41.3%)	30 (81.1%)	72 (39.3%)
Antiviral treatment	1656 (63.0%)	21 (91.3%)	1067 (63.5%)	412 (58.3%)	21 (56.8%)	135 (73.8%)
Glucocorticoids	285 (10.8%)	1 (4.3%)	81 (4.8%)	165 (23.3%)	19 (51.4%)	19 (10.4%)
Intravenous albumin	204 (7.8%)	1 (4.3%)	48 (2.9%)	112 (15.7%)	23 (62.2%)	20 (10.9%)
Chinese medicine	2207 (83.9%)	14 (60.9%)	1420 (84.6%)	607 (85.8%)	23 (62.2%)	143 (78.1%)
Oxygen therapy	1762 (67.0%)	16 (69.6%)	1001 (59.6%)	584 (82.6%)	30 (81.1%)	131 (71.6%)
Mechanical ventilation						
Non-invasive	75 (2.9%)	0 (0%)	7 (0.4%)	32 (4.5%)	20 (54.1%)	16 (8.7%)
Invasive	50 (1.9%)	0 (0%)	5 (0.3%)	18 (2.5%)	14 (37.8%)	13 (7.1%)
Second outcome						
Progress to critical severe	64 (2.4%)	0 (0%)	12 (0.7%)	46 (6.5%)	1 (2.7%)	5 (2.7%)
ICU admission	102 (3.9%)	0 (0%)	8 (0.5%)	43 (6.0%)	32 (86.5%)	19 (10.4%)
Final outcome						
Discharged	2539 (96.5%)	23(100%)	1663 (99.0%)	666 (94.0%)	21 (56.8%)	166 (90.7%)
Dead	62 (2.4%)	0 (0%)	5 (0.3%)	26 (3.7%)	16 (43.2%)	15 (8.2%)
In hospital	29 (1.1%)	0 (0%)	11 (0.7%)	17 (2.4%)	0 (0.0%)	1 (1.1%)
Kidney function laboratory measurements						
Uric acid, μmol/L	277 (222–338)	264 (230–361)	283 (229–341)	269 (209–332)	232 (144–331)	269 (212–329)
Decreased	436 (16.6%)	3 (13.0%)	233 (13.9%)	156 (21.9%)	13 (35.1%)	31 (16.9%)
Normal	1820 (69.2%)	17 (73.9%)	1206 (71.8%)	465 (65.3%)	16 (43.2%)	116 (63.4%)
Elvating	216 (8.2%)	1 (4.3%)	140 (8.3%)	58 (8.1%)	5 (13.5%)	12 (6.6%)
Blood urea nitrogen, mmol/L	4.37 (3.59–5.42)	4.01 (3.17–4.68)	4.3 (3.59–5.20)	4.53 (3.61–5.95)	7.06 (4.82–10.13)	4.42 (3.52–5.48)
Decreased	142 (5.4%)	0 (0%)	88 (5.2%)	40 (5.6%)	1 (2.7%)	13 (7.1%)
Normal	2230 (84.8%)	21 (91.3%)	1458 (86.8%)	597 (83.8%)	19 (51.4%)	135 (73.8%)
Elevating	116 (4.4%)	0 (0%)	43 (2.6%)	47 (6.6%)	13 (35.1%)	13 (7.1%)
Creatinine, μmol/L	64.1 (54.8–75.2)	57.0 (51.7–69.7)	63.9 (55.0–74.8)	64.1 (54.3–76.0)	71.4 (49.85–105.78)	65.1 (55.2–78.1)
Decreased	150 (5.7%)	0 (0%)	74 (4.4%)	56 (7.9%)	9 (24.3%)	11 (6.0%)
Normal	2142 (81.4%)	20 (80.7%)	1426 (84.9%)	548 (77.0%)	14 (37.8%)	134 (73.2%)
Elevating	187 (7.1%)	1 (4.3%)	85 (5.1%)	76 (10.9%)	11(29.7%)	14 (7.7%)
eGFR, ml/(min*1.73m^2^)						
Normal (90–120)	1896 (72.1%)	19 (82.6%)	1264 (75.3%)	476 (66.9%)	18 (48.6%)	119 (65.0%)
Abnormal (15–89)	578 (22.0%)	2 (8.7%)	318 (18.9%)	203 (28.7%)	16 (43.2%)	39 (21.3%)
Light (60–89)	513 (19.5%)	1 (4.3%)	291 (17.3%)	178 (25.0%)	8 (21.6%)	35 (19.1%)
Moderate (30–59)	56 (2.1%)	1 (4.3%)	21 (1.3%)	24 (3.4%)	7 (18.9%)	3 (1.6%)
Severe (15–29)	9 (0.3%)	0 (0%)	6 (0.4%)	1 (0.1%)	1 (2.7%)	1 (0.5%)
Ccr, mL/(min*1.73m^2^)						
Normal (80–120)	1312 (49.9%)	15 (65.2%)	892 (53.1%)	313 (44.0%)	10 (27.0%)	82 (44.8%)
Abnormal (< 79)	631 (24.0%)	1 (4.3%)	406 (24.2%)	183 (25.9%)	7 (18.9%)	34 (18.6%)
Light (51–79)	517 (19.7%)	1 (4.3%)	339 (20.2%)	145 (20.4%)	5 (13.5%)	27 (14.8%)
Moderate (31–50)	92 (3.5%)	0 (0.0%)	55 (3.3%)	32 (4.5%)	1 (2.7%)	4 (2.2%)
Severe (< 30)	22 (0.8%)	0 (0.0%)	12 (0.7%)	6 (0.8%)	1 (2.7%)	3 (1.6%)
EGFR reduction with normal creatinine						
	395 (15.0%)	1 (4.3%)	237 (14.1%)	127 (17.8%)	5 (13.5%)	25 (14.0%)
Ccr reduction with normal creatinine						
	511 (19.4%)	1 (4.3%)	347 (20.7%)	133 (18.7%)	3 (8.1%)	27 (15.1%)
Acute kidney injury						
	26 (0.99%)					
Urine routine on admission						
Proteinuria						
Negative	1616 (61.4%)	11 (47.8%)	1080 (64.3%)	406 (57.0%)	16 (43.2%)	103 (56.3%)
Positive	395 (15.0%)	2 (8.7%)	234 (13.9%)	131 (18.5%)	10 (27.0%)	18 (9.8%)
±	150 (5.7%)	1 (4.3%)	92 (5.5%)	47 (6.6%)	2 (5.4%)	8 (4.4%)
1 +	116 (4.4%)	0 (0%)	76 (4.5%)	34 (4.8%)	3 (8.1%)	3 (1.6%)
2 +	61 (2.3%)	0 (0%)	39 (2.3%)	18 (2.5%)	2 (5.4%)	2 (1.1%)
3 +	68 (2.6%)	1 (4.3%)	27 (1.6%)	32 (4.5%)	3 (8.1%)	5 (2.7%)
Hematuria						
Negative	1796 (68.3%)	13 (56.5%)	1218 (72.5%)	456 (64.0%)	13 (35.1%)	96 (52.5%)
Positive	213 (8.1%)	0 (0%)	94 (5.6%)	81 (11.5%)	13 (35.1%)	25 (13.7%)
±	146 (5.6%)	0 (0%)	69 (4.1%)	55 (7.7%)	4 (10.8%)	18 (9.8%)
1 +	39 (1.5%)	0 (0%)	15 (0.9%)	14 (2.0%)	5 (13.5%)	5 (2.7%)
2 +	19 (0.7%)	0 (0%)	8 (0.5%)	9 (1.3%)	2 (5.4%)	0 (0%)
3 +	9 (0.3%)	0 (0%)	2 (0.1%)	3 (0.4%)	2 (5.4%)	2 (1.1%)
pH value	5.5 (5.0–6.0)	5.5 (5.0–6.0)	5.5 (5.0–6.0)	6.0 (5.0–6.5)	5.5 (5.0–6.0)	6.0 (5.5–6.0)
White blood cell, count/μL	1 (0–7)	6 (0–18)	1 (0–6)	1 (0–7)	4 (0–9)	2 (0–5)

Continuous data was presented as median and interquartile range. Discrete data was presented as counts and percentages

*IQR* interquartile range; *COVID-19* coronavirus disease 2019; *CAD* Coronary artery disease; *COPD* chronic obstructive pulmonary disease; *ICU* Intensive Care Unit; *eGFR* estimated glomerular filtration rate; *Ccr* creatinine clearance rate; *NLR* neutrophil to lymphocyte ratio; *SII* systemic inflammation index

### Kidney function indexes

The levels of uric acid, blood urea nitrogen and serum creatinine increased in 8.2%, 4.4% and 7.1% of the patients, respectively. 22.0% patients showed a decreasing tendency of eGFR, 19.5% of which was a slight decrease, 2.1% a moderate decrease and 0.3% severe decrease. The abnormal rate of eGFR increased along with the severity of disease. Decrease in Ccr was detected in 631 (24.0%) patients, including 517 (19.7%) slight, 92 (3.5%) moderate and 22 (0.8%) severe decrease. On admission, proteinuria and hematuria was found in 15.0% and 8.1% patients, respectively. Both proteinuria and hematuria increased in proportion along with the severity of the disease. It was worth noting that 395 patients with eGFR reduction and 511 patients with Ccr reduction had Scr levels within the normal range. On admission, only 26 patients (0.99%) had an acute kidney injury (AKI) (Table 1).

### Association of kidney function indexes with disease progression in COVID-19 patients

Cox regression analyses were performed to explore the association between each renal function indexes and disease progression. Hematuria, proteinuria, increased uric acid and Scr, and decreased eGFR and Ccr were significantly associated with COVID-19 progression in univariate Cox regression analysis (Additional file 2: Table S2, Additional file 4: Table S4). We next performed a multivariate Cox regression analysis by adjusting age, gender, severe status, heart failure, respiratory failure, total protein, alkaline phosphatase, and neutrophil to lymphocyte (NLR) (Additional file 5: Table S5, Additional file 6: Table S6). Patients with hematuria and proteinuria were 2.38 times (95% CI 1.50–3.78 *P* < 0.001) and 2.16 times (95% CI 1.33–3.51 *P* = 0.002) more likely to have a disease progression, respectively. The elevated baseline of uric acid was associated with disease progression in univariate Cox analysis (HR 1.84, 95% CI 1.09–3.10 *P* = 0.022). However, the association was no longer significant after adjustments for covariates age, gender, severe status, heart failure, respiratory failure, total protein, alkaline phosphatase, and NLR (adjusted HR 1.09, 95% CI 0.83–1.34 *P* = 0.392). BUN and Scr were both significantly associated with disease progression (adjusted HR 3.54, 95% CI 2.36–5.31, *P* < 0.001 and adjusted HR 2.84, 95% CI 1.92–4.21, *P* < 0.001, respectively) (Table 2).

**Table 2:** Association of kidney function indexes with disease progression in COVID-19 patients

Variables	Univariate Cox regression analysis		Multivariate Cox regression analysis	
	HR (95%CI)	*P* value	HR (95%CI)	*P* value
Kidney function laboratory parameters (normal value as reference)				
Hematuria	2.72 (1.81–4.10)	**< 0.001**	2.38 (1.50–3.78)	**< 0.001**
Proteinuria	5.03 (3.34–7.59)	**< 0.001**	2.16 (1.33–3.51)	**0.002**
Elevating uric acid	1.84 (1.09–3.10)	**0.022**	1.09 (0.83–1.34)	0.392
Elevating BUN	7.63 (5.22–11.15)	**< 0.001**	3.54 (2.36–5.31)	**< 0.001**
Elevating Scr	5.28 (3.65–7.63)	**< 0.001**	2.84 (1.92–4.21)	**< 0.001**
Decreasing eGFR	2.40 (1.73–3.34)	**< 0.001**	1.58 (1.07–2.34)	**0.039**
Decreasing Ccr	2.14 (1.22–3.77)	**0.008**	1.90 (0.96–3.77)	0.067

Multivariate Cox regression analysis adjusted for age, gender, severe status, heart failure, respiratory failure, total protein, alkaline phosphatase and neutrophil to lymphocyte ratio (NLR). Bold indicates *P* < 0.05

*BUN* blood urea nitrogen; *Scr* Blood creatinine; *eGFR* estimated glomerular filtration rate; *Ccr* creatinine clearance rate; *AKI* acute kidney injury

Furthermore, we found that eGFR was independently associated with disease progression both in univariate (HR 2.40, 95% CI 1.73–3.34; *P* < 0.001) and multivariate Cox analysis (adjusted HR 1.58, 95% CI 1.07–2.34; *P* = 0.039). Decreasing Ccr showed an adverse effect in univariate analysis (HR 2.14, 95% CI 1.22–3.77 *P* = 0.008), but this association was only marginally significant in multivariate analysis with adjusted HR 1.90 (95% CI 0.96–3.77, *P* = 0.067) (Table 2).

### Association between dynamic changes of kidney function indexes and disease progression in COVID-19 patients

**Fig. 2:**
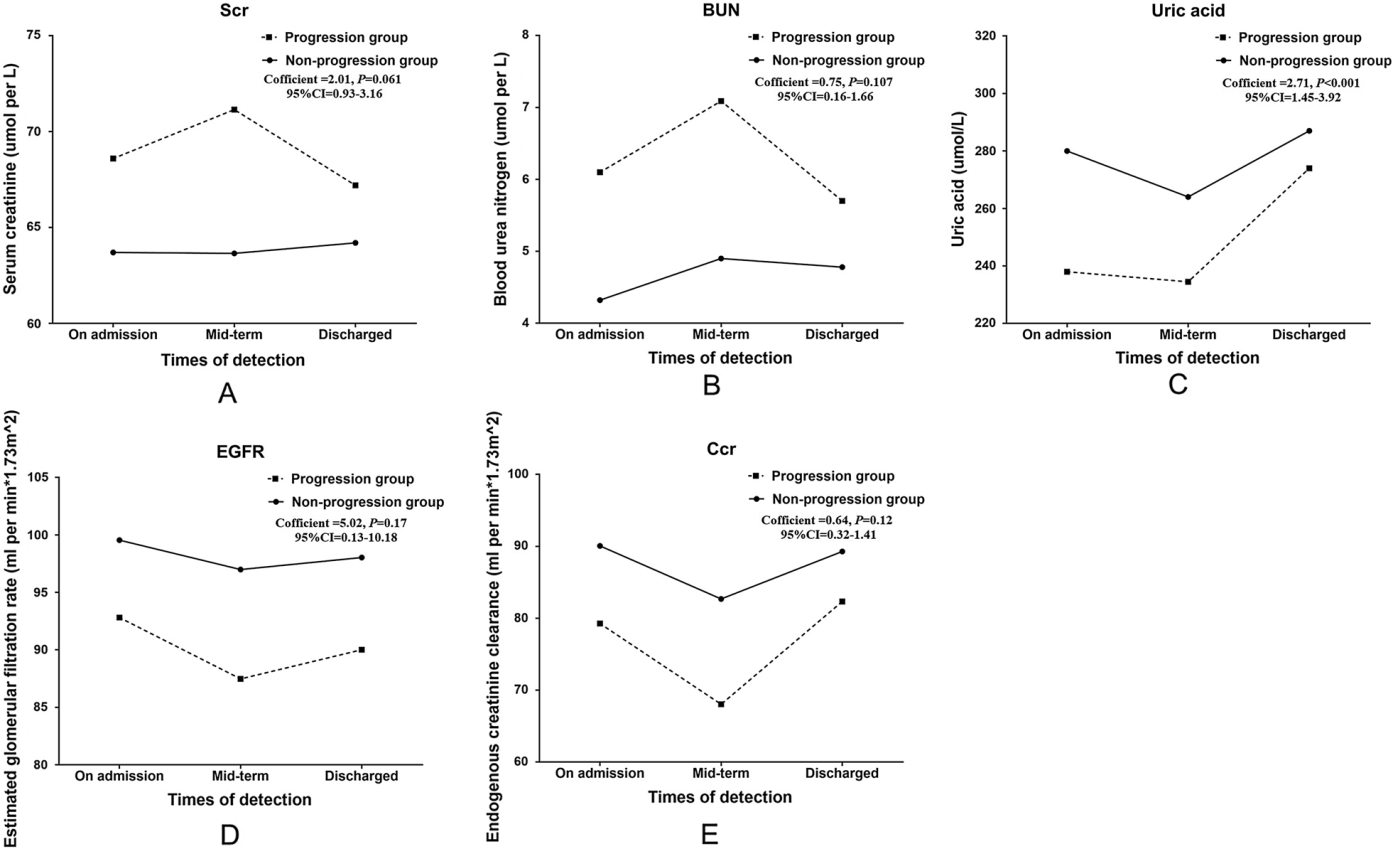
Associations between dynamic changes of kidney function indexes and disease progression in COVID-19 patients. **A** serum creatinine; **B** blood urea nitrogen; **C** uric acid; **D** glomerular filtration rate; **E** endogenous creatinine clearance. solid line indicates non-progression group; dotted line indicates progression group

Kidney function index changes (including uric acid, BUN, Scr, eGFR and Ccr) during hospitalization in COVID-19 patients were shown in Table 3. We next performed a generalized linear mixed model analysis on the dynamic renal function indexes variation at three time points of the clinical course (on admission, mid-term of hospitalization and on discharge). We found that the dynamic changes of uric acid were significantly related to disease progression (Table 4). In details, compared with the non-progression group, Scr and BUN in the progression group stayed at a higher level and were more volatile, which increased rapidly and reached the peak at mid-term of hospitalization. The change of Scr has a bordering statistical significance with the disease progression risk (*P* = 0.061) (Fig. 2A). But the change of BUN was not associated with the disease progression risk (*P* = 0.107) (Fig. 2B). The uric acid level in both groups declined in mid-term and increased on discharge. However, the variation trend of uric acid was significantly different between two groups: patients in progression group tended to have a significantly lower level at the three time points (*P* < 0.001) (Fig. 2C).

**Table 3:** Kidney function index changes during hospitalization in COVID-19 patients

Variables	On admission to hospital		During hospital stay		Discharge	
	Progression	Non-progression	Progression	Non-progression	Progression	Non-progression
Elevating uric acid	17 (11.0%)	199 (8.6%)	17 (16.0%)	42 (8.5%)	20 (16.3%)	96 (9.7%)
Elevating BUN	39 (25.3%)	77 (3.3%)	40 (38.5%)	34 (7.5%)	56 (45.5%)	44 (4.4%)
Elevating Scr	40 (24.5%)	147 (6.3%)	35 (33.0%)	57 (11.6%)	35 (28.5%)	99 (9.9%)
Decreasing eGFR	65 (39.9%)	513 (20.8%)	52 (42.5%)	153 (31.2%)	54 (44.3%)	274 (27.5%)
Decreasing Ccr	24 (49.0%)	607 (32.0%)	8 (57.1%)	88 (43.4%)	13 (44.8%)	151 (34.9%)

Data presented was count and percentage

*BUN* blood urea nitrogen; *Scr* blood creatinine; *eGFR* estimated glomerular filtration rate; *Ccr* creatinine clearance rate

**Table 4:** Generalized linear mixed analysis of the association between dynamic variation of kidney function indexes and disease progression during hospitalization in COVID-19 patients

Kidney indicators	F value	Coefficient	95% CI of coefficient	P value
Scr	3.78	2.01	0.93–3.16	**0.061**
BUN	2.60	0.75	0.16–1.66	0.107
Uric acid	17.70	2.71	1.45–3.92	**< 0.001**
eGFR	1.15	5.02	0.13–10.18	0.170
Ccr	2.10	0.64	0.32–1.41	0.120

Generalize linear mixed analysis adjusted for age, gender,severe status, complications (heart failure, respiratory failure) and laboratory testing indicators on admission including total protein, alkaline phosphatase and neutrophil to lymphocyte ratio

Bold indicates *P* < 0.05

*Scr* Blood creatinine, *BUN* blood urea nitrogen; *eGFR* estimated glomerular filtration rate; *Ccr* creatinine clearance rate; *COVID-19* coronavirus disease 2019

The early kidney indicator, eGFR, showed no significantly different variation trend between two groups (*P* = 0.170) (Fig. 2D). Patients in progression group showed a lower level of eGFR on admission, then dropped significantly on mid-term, and gradually recovered but remained in a lower level than that in non-progression group on discharge. Similarly, the variation trend of Ccr seemed not to be associated with disease progression (*P* = 0.120) (Fig. 2E).

## Discussion

In this study, we found there was a high proportion of early kidney function injury in COVID-19 patients on admission. eGFR and Ccr decreased in 22.0% and 24.0% of patients, respectively. This study also demonstrated a significant association between early kidney function injury and poor outcomes in patients with COVID-19 (Additional file 3: Table S3).

Because the SARS-CoV-2 spread mainly through the respiratory airway with viral pneumonia symptoms as the first onset and most typical symptoms, clinicians have paid considerable attention on the pneumonia treatment. However, the expression level of ACE2 in kidney, especially in the renal tubules, was no less than that in lung, suggesting that kidney might also be another important target organ for SARS-CoV-2 virus [11]. Evidence on Middle East respiratory syndrome coronavirus (MERS-CoV) indicated that SARS-CoV-2 may also attack directly at renal tubular epithelial cells [12]. The granules of SARS-CoV-2 have been isolated in patients' urine. Viral particles were also detected in electron micrographs of kidneys by autopsy, supporting the direct viral attack theory in kidney [13]. Although, the mechanism of kidney damage by SARS-CoV-2 has not yet been fully elucidated and further research is warranted to investigate these issues.

Our study focused on the association of early kidney injury with clinical outcomes of COVID-19. We revealed that patients with normal baseline serum creatinine have showed the decrease of eGFR and Ccr on early stage. Our results were consistent with findings from a previous study that none of 12 COVID-19 patients have abnormal BUN and Scr levels, but the abnormal reduction rates of eGFR and Ccr was 66.7% and 41.7%, respectively [14]. We found that 22.0% and 24.0% of patients had decreasing eGFR and Ccr on admission respectively, and these two ratios were markedly less than those reported by the previous study. The possible reason may be that our study had a larger sample and included more representative patients. Our finding indicated that early kidney dysfunctions was found in a large number of COVID-19 patients on admission, even in mild/moderate patients. Accumulated evidence supported that it was more likely for those patients who died from COVID-19 to have experienced acute kidney injury (25%) during hospitalization [15], and acute kidney injury was associated with death risk [16]. However, previous studies have mainly focused on acute kidney injury, but neglected the early kidney function dysfunction decrease (such as decrease of eGFR or Ccr). Early detection and intervention of renal dysfunction might effectively prevent the development of severe acute kidney injury. Our study demonstrated that the early kidney injury indicator, eGFR, was an independent risk factor for the poor outcomes in patients with COVID-19. In addition, it should be pointed out that patients with a normal range level of BUN and Scr on admission had an abnormal decrease eGFR. Thus, eGFR could be a more sensitive index than BUN, Scr, and acute kidney injury in early warning of adverse prognosis.

Hematuria and proteinuria are common indicators of abnormal urinary analysis and have been increasingly recognized as important markers in attack glomerular and podocytes [17]. A study on 710 COVID-19 patients reported that 44% of patients have proteinuria and 26.9% hematuria on admission [5]. Researchers also found that urinalysis can detect the early renal-impairment in patients with COVID-19 [18]. But their clinical values and effects on disease progression were not yet known. In our analysis, both hematuria and proteinuria on admission were independent risk factors for the progression of COVID-19.

A study based on 138 hospitalized COVID-19 patients revealed the incidence of elevated Scr and BUN was 15.5% and 14.1%, respectively [5, 19]. Kidney function dynamic monitoring data showed that 31%, 22% and 20% patients with increased BUN, Scr and uric acid levels, respectively [20]. Another study revealed that the median levels of Scr and BUN were 77 μmol/L and 5.7 mmol/L, with the elevated in 14.4% and 13.1% COVID-19 patients, respectively. Our findings showed that 7.1% and 4.4% patients had elevated Scr (median level 64.1 μmol/L) and BUN (4.37 mmol/L), respectively. We also found that BUN and Scr were both risk factors for disease progression, which was consistent with the result of Cheng [3]. In addition, the dynamic changes of Scr and uric acid reflected the changes of kidney injury in the clinical process. Our generalized linear mixed model analysis indicated that the dynamic trajectories of uric acid was significantly related to disease progression and the variation of Scr deserved continuous exploration. Therefore, dynamic monitoring of uric acid levels will be helpful to predict the prognosis of patients and take timely treatment measures.

Uric acid, BUN, and Scr are the blood biochemistry indexes directly reflecting kidney function. These indicators are convenient to obtain and are helpful for clinicians to monitor the kidney function of patients with COVID-19. Though the decline of eGFR on admission is related to poor prognosis, the evaluation of eGFR needs to be carried out according to the characteristics of each patient. The Uric acid variation seem more suitable to predict disease progression during hospitalization. Considering that these indicators may play a different role in various disease stages of COVID-19, therefore all of them were analyzed in our study.

Currently, most clinicians adopted the definition of AKI recommended by the Organization for Improving the Prognosis of Kidney Diseases Worldwide (KDIGO) as one of the following: (1) an increase in serum creatinine by ≥ 0.3 mg/dL (≥ 26.5 µmol/L) within 48 h, (2) an increase in serum creatinine to ≥ 1.5 times baseline within the previous 7 days, (3) urine volume ≤ 0.5 mL/kg/h for 6 h [10]. Due to the special nature of the Huoshenshan hospital and the urgency of the epidemic in Wuhan at that time, we cannot obtain the baseline levels of the patient's blood creatinine and the patient's urine output. Therefore, we identified the AKI patients on admission according to the special situation of AKI diagnosis issued by KDIGO: endogenous creatinine clearance < 60 mL/min or serum creatinine > 133umol/L, BUN > 20 mmol/L [10]. We then found 26 patients with acute kidney injury. According to this criterion, we probably missed some patients with a large increase in serum creatinine in a 48 h after admission or a significant decrease in urine volume, because some of the AKI occurred in a short period and recovered with the start of treatment [21]. This may be the main reason why we only observed early AKI in 26 patients at admission, which could lead to underestimate of the AKI incidence in our study. However, our primary purpose was to explore the association of early kidney function indexes and their dynamic changes with disease progression of COVID-19. Therefore, we did not analyze the association of AKI with disease progression.

Even though this cohort study enrolled 2630 hospitalized patients, there were several limitations. First, the clinical data came from a single center, and our findings need to be validated by external cohorts. Second, our study was a real-world study with dataset from clinical practice, it was difficult to avoid information bias caused by missing and inaccurate clinical information. Third, although we attempted to adjust for potential confounders in multivariate analysis, there might be unmeasured or unknown confounding bias in our study.

## Conclusion

There was a high proportion of early kidney function injury in COVID-19 patients on admission. The early injury in kidney function predicted the poor prognosis of COVID-19. The dynamic changes blood uric acid were associated the poor prognosis. Our findings could help clinicians prevent worse outcome and promote the recovery of patients.

## Supplementary Information

**Additional file 1: Table S1.** Upper and lower limits of kidney function laboratory parameters in male and female patients with COVID-19.

**Additional file 2: Table S2.** Risk factors associated with disease progression in COVID-19 patients in univariate Cox regression analysis.

**Additional file 3: Table S3.** Summary and comparison of recent studies on kidney damage from COVID-19 patients.

**Additional file 4: Table S4.** Risk factor associated disease progression in COVID-19 patients in univariate Cox regression analysis.

**Additional file 5: Table S5.** Collinearity diagnosticsof variables in multivariate analysis.

**Additional file 6: Table S6.**Associations of kidney function indexes with disease progression in COVID-19 patients by multivariate cox regression analyses.

## Data Availability

The datasets used and/or analysed during the current study are available from the corresponding author on reasonable request.

## Abbreviations

COVID-19: Coronavirus disease 2019
SARS-CoV-2: Severe respiratory syndrome coronavirus 2
eGFR: Estimated glomerular filtration rate
Ccr: Creatinine clearance rate
Scr: Serum creatinine
BUN: Blood urea nitrogen
AKI: Acute kidney injury
UA: Urine acid
CRP: C-reactive protein
BNP: Brain natriuretic peptide
NLR: Neutrophil-to-lymphocyte ratio
KDIGO: Kidney Disease Improving Global Outcomes
ICU: Intensive Care Unit
VIF: Variance inflation factor
HRs: Hazard ratios
CIs: Confidence intervals
IQR: Interquartile range
ACE2: Angiotensin-converting enzyme 2
