# Supplementary material for: Early kidney injury predicts disease progression in patients with COVID-19: a cohort study
Source: BMC Infect Dis. 2021 Sep 27;21:1012. doi: 10.1186/s12879-021-06576-9 (PMC8474921; doi:10.1186/s12879-021-06576-9)
Supplement: Supplementary file 1 — Additional file 1: Table S1. Upper and lower limits of kidney function laboratory parameters in male and female patients with COVID-19. [file 12879_2021_6576_MOESM1_ESM.docx]

| **Supplementary Table 1. Upper and lower limits of kidney function laboratory parameters in male and female patients with COVID-19** | | | | | | | | | | | | | |
| --- | --- | --- | --- | --- | --- | --- | --- | --- | --- | --- | --- | --- | --- |
| Kidney  indexes | Men | | | | | |  | Woman | | | | | |
|  | < 60  years old | | 60 ~ 80  years old | | > 80  years old | |  | < 60  years old | | 60 ~ 80  years old | | > 80  years old | |
|  | U | L | U | L | U | L |  | U | L | U | L | U | L |
| Scr,  μmol/L | 97.0 | 57.0 | 111.0 | 57.0 | 73.0 | 41.0 |  | 73.0 | 41.0 | 81.0 | 41.0 | 73.0 | 41.0 |
| BUN,  mmol/L | 8.0 | 3.1 | 9.5 | 3.6 | 7.5 | 2.6 |  | 7.5 | 2.6 | 8.8 | 3.1 | 7.5 | 2.6 |

U, upper limit; L, lower limit; Scr, Blood creatinine; BUN, blood urea nitrogen; COVID-19, coronavirus disease 2019.
