# Supplementary material for: Early kidney injury predicts disease progression in patients with COVID-19: a cohort study
Source: BMC Infect Dis. 2021 Sep 27;21:1012. doi: 10.1186/s12879-021-06576-9 (PMC8474921; doi:10.1186/s12879-021-06576-9)
Supplement: Supplementary file 2 — Additional file 2: Table S2. Risk factors associated with disease progression in COVID-19 patients in univariate Cox regression analysis. [file 12879_2021_6576_MOESM2_ESM.docx]

| **Supplementary Table 2. Risk factors associated with disease progression in COVID-19 patients in univariate Cox regression analysis** | | |
| --- | --- | --- |
| Variables | Univariate Cox regression analysis | |
|  | HR (95%CI) | *P* value |
| **Age (years)** |  |  |
| > 60 | 3.77 (2.59-5.50) | **<0.001** |
| **Gender** |  |  |
| Male | 1.75 (1.27-2.41) | **0.001** |
| **Symptoms** |  |  |
| Dyspnea | 1.45 (1.06-1.98) | **0.019** |
| Myalgia | 1.43 (1.03-1.99) | **0.034** |
| **Types of symptoms** |  |  |
| > 3 | 1.33 (0.98-1.81) | 0.069 |
| **Comorbidities** |  |  |
| Hypertension | 2.15 (1.58-2.92) | **<0.001** |
| Coronary artery Disease | 2.31 (1.47-3.63) | **<0.001** |
| Heart failure | 12.28 (5.03-29.98) | **<0.001** |
| Other cardiovascular disease | 4.54 (2.81-7.33) | **<0.001** |
| Respiratory distress | 17.37 (8.13-37.14) | **<0.001** |
| COPD | 4.64 (2.05-10.49) | **<0.001** |
| Respiratory failure | 16.79 (10.50-26.86) | **<0.001** |
| Chronic kidney disease | 3.91 (1.83-8.35) | **<0.001** |
| Tumor history | 3.69 (1.63-8.34) | **0.002** |
| Diabetes | 1.34 (0.90-2.00) | 0.145 |
| Respiratory comorbidity | 1.73 (1.53-1.96) | **<0.001** |
| Other comorbidities | 1.69 (1.22-2.33) | **<0.001** |
| Comorbidity counts (>3) | 3.50 (2.12-5.79) | **<0.001** |
| **Disease status** |  |  |
| Severe (other status as reference) | 8.32 (5.83-11.89) | **<0.001** |
| **Other laboratory measurements (normal value as reference)** | | |
| NLR | 10.07 (6.96-14.56) | **<0.001** |
| ALP | 1.92 (1.26-2.93) | **0.002** |

Bold indicates *P*< 0.05.

COVID-19, coronavirus disease 2019; COPD, chronic obstructive pulmonary disease; BUN, blood urea nitrogen; Scr, Blood creatinine; eGFR, estimated glomerular filtration rate; Ccr, creatinine clearance; AKI, acute kidney injury; NLR, neutrophil to lymphocyte ratio; ALP, alkaline phosphatase.
