# Supplementary material for: Early kidney injury predicts disease progression in patients with COVID-19: a cohort study
Source: BMC Infect Dis. 2021 Sep 27;21:1012. doi: 10.1186/s12879-021-06576-9 (PMC8474921; doi:10.1186/s12879-021-06576-9)
Supplement: Supplementary file 3 — Additional file 3: Table S3. Summary and comparison of recent studies on kidney damage from COVID-19 patients. [file 12879_2021_6576_MOESM3_ESM.doc]

| **Table S3** Summary and comparison of recent studies on kidney damage from COVID-19 patients. | | | | | | |
| --- | --- | --- | --- | --- | --- | --- |
| Authors | Published time | Country | Sample size | Study design | Exposure | Outcome |
| Our study | | China | 2,630  patients | Retrospective cohort study | eGFR, Ccr, proteinuria, hematuria, Scr, Bun | Disease progression risk including death, transfer to ICU, and severity aggravation |
| Jose Portolés[1] | 2020.08 | Spain | 1603 patients | Prospective  cohort study | Haematuria, Scr,  acute kidney injury,  chronic kidney disease | In-hospital death |
| Jia H Ng[2] | 2021.02 | New York | 9,657 patients | Retrospective cohort study | Acute kidney injury stage 1, 2, and 3 | In-hospital death, requiring dialysis at discharge,  recovery of kidney function. |
| Guangchang Pei [3] | 2020.06 | China | 333 patients | Retrospective cohort study | kidney complications | Death in follow-up period |
| Jeong-Hoon Lim[4] | 2020.06 | South Korea | 164 patients | Retrospective cohort study | Acute kidney injury stage 1, 2, and 3 | In-hospital  death |

**References:**

1 Farouk SS, Fiaccadori E, Cravedi P, Campbell KN: COVID-19 and the kidney: What we think we know so far and what we don't. J NEPHROL 2020;33:1213-1218.

2 Basile DP, Anderson MD, Sutton TA: Pathophysiology of acute kidney injury. COMPR PHYSIOL 2012;2:1303-1353.

3 Portolés J, Marques M, López-Sánchez P, de Valdenebro M, Muñez E, Serrano ML, Malo R, García E, Cuervas V: Chronic kidney disease and acute kidney injury in the COVID-19 Spanish outbreak. Nephrol Dial Transplant 2020;35:1353-1361.

4 Ng JH, Hirsch JS, Hazzan A, Wanchoo R, Shah HH, Malieckal DA, Ross DW, Sharma P, Sakhiya V, Fishbane S, Jhaveri KD: Outcomes among patients hospitalized with COVID-19 and acute kidney injury. AM J KIDNEY DIS 2021;77:204-215.
