# Supplementary material for: Early kidney injury predicts disease progression in patients with COVID-19: a cohort study
Source: BMC Infect Dis. 2021 Sep 27;21:1012. doi: 10.1186/s12879-021-06576-9 (PMC8474921; doi:10.1186/s12879-021-06576-9)
Supplement: Supplementary file 4 — Additional file 4: Table S4. Risk factor associated disease progression in COVID-19 patients in univariate Cox regression analysis. [file 12879_2021_6576_MOESM4_ESM.doc]

| **Table S4.** Risk factor associated disease progression in COVID-19 patients in univariate Cox regression analysis | | |
| --- | --- | --- |
|  | Univariate Cox regression analysis | |
| Variables | HR (95%CI) | *P* value |
| Fever on admission | 1.07（0.74-1.54） | 0.731 |
| Traditional Chinese medicine | 0.71（0.47-1.08） | 0.110 |

Bold indicates *P* < 0.05.
