# Supplementary material for: Early kidney injury predicts disease progression in patients with COVID-19: a cohort study
Source: BMC Infect Dis. 2021 Sep 27;21:1012. doi: 10.1186/s12879-021-06576-9 (PMC8474921; doi:10.1186/s12879-021-06576-9)
Supplement: Supplementary file 5 — Additional file 5: Table S5. Collinearity diagnosticsof variables in multivariate analysis. [file 12879_2021_6576_MOESM5_ESM.doc]

| **Table S5.** Collinearity diagnostics of variables in multivariate analysis | | | |
| --- | --- | --- | --- |
| Variables | Eigenvalue | Condition index | Variance inflation factor |
| Age | 7.841 | 1.000 | 1.081 |
| Gender | 1.025 | 2.765 | 1.028 |
| Severe status | 0.739 | 3.257 | 1.054 |
| Heart failure | 0.614 | 3.575 | 1.047 |
| Respiratory failure | 0.312 | 5.016 | 1.146 |
| Total protein | 0.225 | 5.903 | 2.110 |
| Alkaline phosphatase | 0.127 | 7.853 | 2.189 |
| Neutrophil to lymphocyte ratio | 0.106 | 9.338 | 2.009 |
| Though time from diagnosis | 0.070 | **10.614** | **5.099** |
| Body temperature at the admission | 0.041 | **13.843** | **5.189** |
| Antibiotics treatment | 0.007 | **34.652** | **5.069** |
| Antiviral treatment | 0.000 | **276.143** | **5.050** |

The Condition index >10 and VIF >5 are considered as collinearity and can not entered the multivariate analysis.
