# Supplementary material for: Early kidney injury predicts disease progression in patients with COVID-19: a cohort study
Source: BMC Infect Dis. 2021 Sep 27;21:1012. doi: 10.1186/s12879-021-06576-9 (PMC8474921; doi:10.1186/s12879-021-06576-9)
Supplement: Supplementary file 6 — Additional file 6: Table S6.Associations of kidney function indexes with disease progression in COVID-19 patients by multivariate cox regression analyses. [file 12879_2021_6576_MOESM6_ESM.docx]

| **Table S6. Associations of kidney function indexes with disease progression in COVID-19 patients by multivariate cox regression analyses.** | | | | | | |
| --- | --- | --- | --- | --- | --- | --- |
| Variables | Model 1 | | Model 2 | | Model 3 | |
|  | HR (95%CI) | *P* value | HR (95%CI) | *P* value | HR (95%CI) | *P* value |
| **Kidney function laboratory parameters (normal value as reference)** | | | | | | |
| Hematuria | 2.37 (1.54-3.65) | **<0.001** | 1.64 (1.05-2.56) | **0.021** | 2.38 (1.50-3.78) | **<0.001** |
| Proteinuria | 4.08 (2.64-6.30) | **<0.001** | 2.37 (1.49-3.76) | **<0.001** | 2.16 (1.33-3.51) | **0.002** |
| Elevating uric acid | 2.84 (2.00-4.02) | **<0.001** | 1.34 (1.12-1.62) | **0.002** | 1.09 (0.83-1.34) | 0.392 |
| Elevating BUN | 5.57 (3.77-8.29) | **<0.001** | 3.27 (2.17-4.93) | **<0.001** | 3.54 (2.36-5.31) | **<0.001** |
| Elevating Scr | 3.15 (2.10-4.72) | **<0.001** | 2.40 (1.59-3.65) | **<0.001** | 2.84 (1.92-4.21) | **<0.001** |
| Decreasing eGFR | 1.48 (1.03-2.12) | **0.036** | 1.42 (0.98-2.07) | 0.065 | 1.58 (1.07-2.34) | **0.039** |
| Decreasing Ccr | 1.31 (0.67-2.55) | 0.432 | 1.87 (0.96-3.63) | 0.065 | 1.90 (0.96-3.77) | 0.067 |

Model 1was adjusted for age, gender.

Model 2 was additionally adjusted for severity of COVID-19, heart failure, and respiratory failure,based on Model 1.

Model 3 was additionally adjusted total protein, alkaline phosphatase and neutrophil to lymphocyte ratio (NLR), based on Model 2.

Bold indicates *P* < 0.05.

Abbreviation: BUN, blood urea nitrogen; Scr, Blood creatinine; eGFR, estimated glomerular filtration rate; Ccr, creatinine clearance; AKI, acute kidney injury.
